# An equitable vaccine delivery system: Lessons from the COVID-19 vaccine rollout in Canada

**DOI:** 10.1371/journal.pone.0279929

**Published:** 2022-12-30

**Authors:** Ksenia Kholina, Shawn H. E. Harmon, Janice E. Graham

**Affiliations:** 1 Department of Pediatrics (Infectious Diseases), Faculty of Medicine, Dalhousie University, Halifax, NS, Canada; 2 Technoscience & Regulation Research Unit, Faculty of Medicine, Dalhousie University, Halifax, NS, Canada; Universidade Federal de Minas Gerais, BRAZIL

## Abstract

**Background:**

The COVID-19 pandemic exacerbated existing health disparities and disproportionately affected vulnerable individuals and communities (e.g., low-income, precariously housed or in institutional settings, racialized, migrant, refugee, 2SLBGTQ+). Despite their higher risk of infection and sub-optimal access to healthcare, Canada’s COVID-19 vaccination strategy focused primarily on age, as well as medical and occupational risk factors.

**Methods:**

We conducted a mixed-methods constant comparative qualitative analysis of epidemiological data from a national database of COVID-19 cases and vaccine coverage in four Canadian jurisdictions. Jurisdictional policies, policy updates, and associated press releases were collected from government websites, and qualitative data were collected through 34 semi-structured interviews of key informants from nine Canadian jurisdictions. Interviews were coded and analyzed for themes and patterns.

**Results:**

COVID-19 vaccines were rolled out in Canada in three phases, each accompanied by specific challenges. Vaccine delivery systems typically featured large-venue mass immunization sites that presented a variety of barriers for those from vulnerable communities. The engagement and targeted outreach that featured in the later phases were driven predominantly by the efforts of community organizations and primary care providers, with limited support from provincial governments.

**Conclusions:**

While COVID-19 vaccine rollout in Canada is largely considered a success, such an interpretation is shaped by the metrics chosen. Vaccine delivery systems across Canada need substantial improvements to ensure optimal uptake and equitable access for all. Our findings suggest a more equitable model for vaccine delivery featuring early establishment of local barrier-free clinics, culturally safe and representative environment, as well as multi-lingual assistance, among other vulnerability-sensitive elements.

## Introduction

COVID-19 was first detected in late January 2020 in Canada associated with international travel [1]. Subsequent epidemiological surveillance showed that infection rates and disease outcomes differed by demographic characteristics, largely exacerbating existing health disparities [1–3]. Despite calls to collect, aggregate and release race-based data, the availability of such data remained limited [1, 4], with the notable exception of Ontario [5]. Consequently, analyses largely relying on geographic estimations that show that racialized neighbourhoods experienced disproportionally high rates of infection and mortality from COVID-19 due to a variety of socioeconomic risk factors (e.g., crowded housing, overrepresentation in high-risk essential occupations), and barriers to accessing healthcare [2, 3, 6–10]. COVID-19 exacerbated systemic intersectional discrimination and amplified existing vulnerabilities of marginalized communities [7, 11, 12] but consideration of social determinants of health largely remained absent from public health modelling and decision-making [13].

In light of these disparities, the COVID-19 pandemic can be viewed through the closely associated lenses of ‘vulnerability’ and ‘risk’ [14–16] which have been used to analyze many disasters and disaster-recovery efforts during the last five decades:

Vulnerability is the potential harm incurred by a person, asset, activity or assemblage of items that is at risk… [T]he risk is motivated by natural, technological, social, intentional, or complex hazards and the potential outcome is a disaster. As it is mainly the result of social, economic, political, and cultural factors in decision-making, vulnerability is constructed socially [17].

Vulnerability is shaped by a multiplicity of systems of oppression, including white supremacy, anti-Black and Indigenous racism, capitalism and classism. The US Centers for Disease Control and Prevention (CDC) developed a ‘social vulnerability index’ as an aid to disaster management, organizing census factors by theme: Socioeconomic Status (income, poverty, employment, and education variables); Household Composition (age, single-parenting, and disability variables); Minority Status (race, ethnicity, and English language proficiency variables); Housing/Transportation (housing structure, crowding, and vehicle access variables) [18]. Just one of many ‘vulnerability models’ developed to assess susceptibility to loss and harm, this index identifies obstacles to recovery and critique disaster decision-making and interventions [19].

Vulnerability is the confounding of the capabilities of people to foresee—and take action to avoid—danger, often as a result of limitations on access to critical political, economic, social, human, physical, and natural resources [19]. Vulnerability therefore arises when political, social, and economic structures deny people an environment within which their *capacities* can be operationalized as *capabilities* [20]. The process can take many forms, including ‘marginalization’, which relegates people to voicelessness, powerlessness, and invisibility through dismissal of their knowledge and neglect of their interests [21]. Often the central role played by governments (and powerful capitalist organizations to which they are allied) in generating the conditions of risk (e.g., income disparity, maldevelopment, habitat destruction, infrastructure inadequacy) is often ignored or downplayed [22]. In short, neither scholarship nor policy-making effectively address ‘risk-creation’ and the role of governments [23]. This is a particularly damaging—even deadly—blind spot.

In this paper, we examine the COVID-19 vaccine rollout in Canada through the lens of vulnerability, focusing on vaccination strategies and tactics adopted in Alberta, Ontario, Nova Scotia, and Yukon. We highlight some of the ways by which these governments were complicit in creating or ignoring the blockages to vulnerability-reducing capabilities that Wisner has identified as contributing to marginalization [23]. In short, we assess Canada’s COVID-19 vaccine rollout with a view to its role in risk-creation. First, we describe our methods. Second, we outline the phases of the vaccine rollout, and the prioritization strategies that informed them, offering a timeline of access to vaccines. Third, drawing on expert interviews, we describe some of the challenges faced by public health decision-makers, and efforts employed to overcome them. We conclude with some evidence-informed recommendations on how to more equitably deliver vaccines with the larger goal of diminishing systems’ contributions to vulnerability.

## Methods

This study received ethical approval from the IWK Health Centre Research Ethics Board (#1025970) and utilized a mixed-methods constant comparative approach. This involved i) analysis of COVID case trends coincident with the provincial or territorial health policies in place to determine associations, and ii) 34 key-informant interviews. Key policies from four Canadian jurisdictions—Alberta, Ontario, Nova Scotia, and Yukon—were analyzed in parallel with epidemiological data, news releases and press briefings, and our qualitative interview data [24]. These jurisdictions were selected for their known differences in pandemic experiences linked to multiple factors, including geography, population density, demographics, socio-cultural characteristics, economic conditions, and political climate. For each jurisdiction, data on COVID-19 cases, deaths, and vaccine uptake were extracted from the federal Public Health Infobase [25] for the period from January 31, 2020, to October 5, 2021. Policy updates related to pandemic public health measures (e.g., lockdowns, travel restrictions, business closures and openings) and vaccine rollout (e.g., vaccine eligibility and availability) were accessed through respective provincial and territorial government news releases [26–29]. Pertinent policies were plotted on graphs depicting disease burden and interpreted with the aid of news releases and press briefings [30]. The qualitative component draws on 34 semi-structured key-informant interviews conducted via Zoom of public health practitioners, frontline healthcare providers, scholars and union leaders from September to December 2021, recruited from across Canada using a combination of purposive and snowball sampling. Written consent was obtained from all participants. All interviews were completed in one session that ranged between 31 minutes and 1 hour and 13 minutes in length. The interviews were audio and video recorded. Transcripts were produced and edited using Otter, an online artificial intelligence software, and coded and analyzed in NVivo. A structured three-stage team-based coding approach was employed [31] involving initial coding (in most cases, done by the lead interviewer), joint coding (by the first coder and the reviewer), and consistency coding (by the reviewer). Data were analyzed using Braun & Clarke’s thematic analysis approach [32]. Codes were inductively organized into categories under emerging themes into a thematic network [33] finalized by the project team and associated with pertinent literature. All epidemiological, policy and informant interview data were triangulated using constant comparative analysis [24]. The analysis yielded a rich dataset; this article primarily addresses themes related to vaccine rollout while other findings are discussed elsewhere [e.g., 30].

## Findings

Falling into one of four groups (public health officials (PH; n = 18); frontline healthcare workers (FL; n = 8); healthcare union leaders (U; n = 3); and health scholars (S; n = 5)), study interviewees represented one or more of nine jurisdictions: Global (n = 3); Federal (n = 5); Ontario (n = 10); Alberta (n = 4); Saskatchewan (n = 3); British Columbia (n = 2); Northwest Territories (n = 2); Nova Scotia (n = 2); New Brunswick (n = 1); Prince Edward Island (n = 1); Quebec (n = 1).

In November 2020, prior to COVID-19 vaccine availability, the Advisory Committee on Immunization (NACI) recommended prioritizing the elderly, individuals with pre-existing conditions, and healthcare and essential workers [34], a ranking approach similar to those adopted in other high-income countries [35]. A notable feature of the NACI framework was the stated prioritization of those living or working in environments with elevated risks of transmission or disproportionate consequences of infection (i.e., Indigenous communities, over which the federal government has primary healthcare responsibility pursuant to s 91(X) of the *Constitution Act 1867* and its fiduciary duties under the honour of the Crown) [34]. While NACI is a national advisory body, vaccine delivery in Canada is primarily a provincial/territorial responsibility. As a result, NACI recommendations met with variable implementation across the country [36, 37]. In most jurisdictions, priority vaccination was largely based on age, a limited number of chronic medical conditions, and healthcare occupation despite the known impact of socio-demographic factors such as ethnicity and income [36]. See *S1 Table: Prioritization of Populations (Supporting Information)* for the NACI-recommended priorities and access timelines adopted in the jurisdictions.

### COVID-19 vaccine rollouts: By the epidemiological numbers

**Fig 1:**
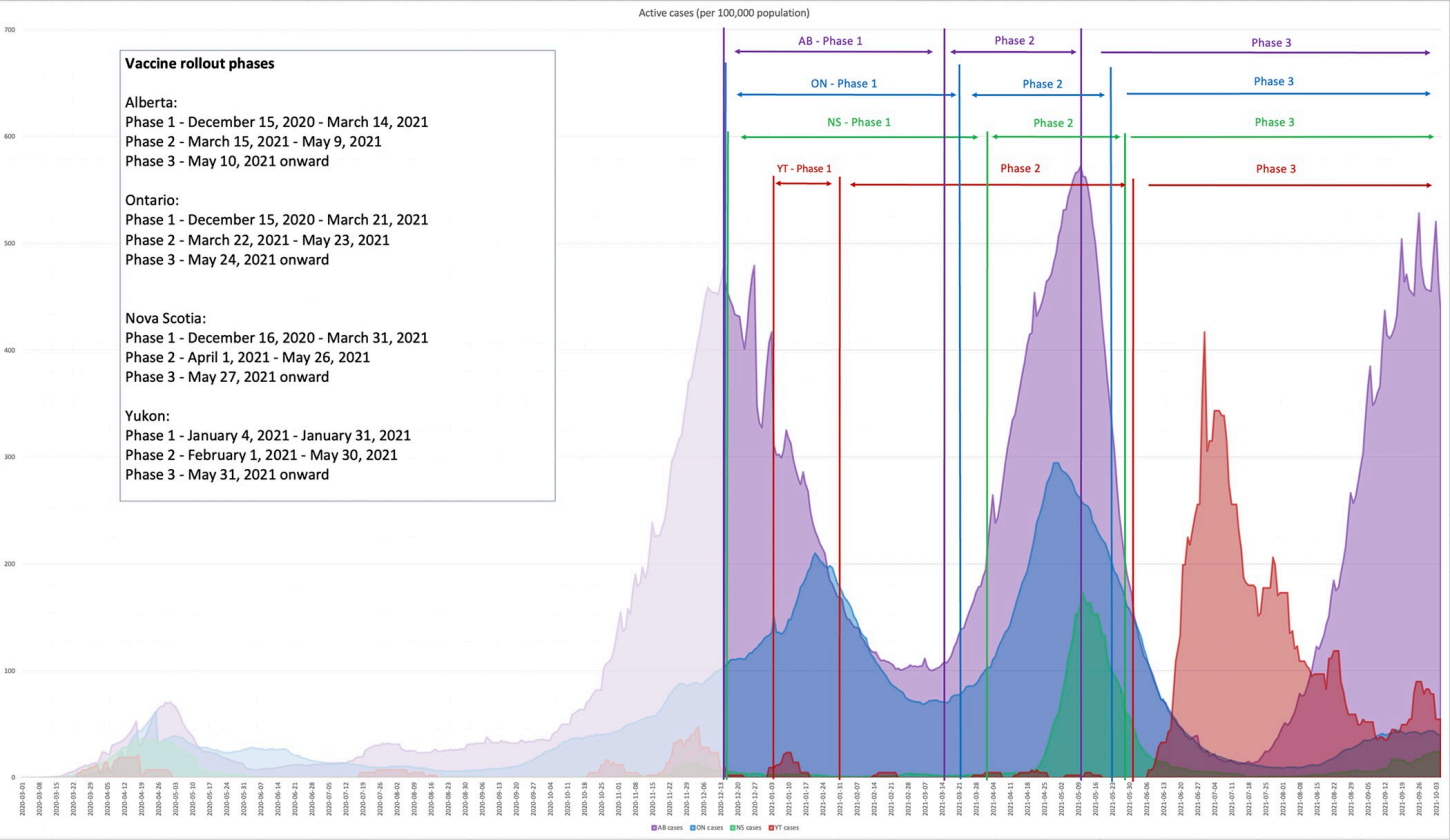
Vaccine rollout phases by province. COVID-19 epidemiology: active cases by 100,000 population in Alberta, Ontario, Nova Scotia and Yukon (March 1, 2020 –October 5, 2021). Three phases of vaccine rollout are indicated.

We situated vaccine implementation in the context of COVID-19 disease epidemiology and public health measures deployed in Alberta, Ontario, Nova Scotia and Yukon. While COVID-19 infection rates, public health measures and outcomes differed substantially across Canadian communities [1, 38], the pandemic is typically described in ‘waves’ [39]. Vaccine rollout began in the middle of the Second Wave in most jurisdictions [39], employing a three-phase approach, generally starting with the highest priority groups outlined in the NACI recommendations [34], with some variation across the country. *Fig 1: Vaccine Rollout Phases by Province* depicts the Phases against active cases per 100,000 population over time for Alberta, Ontario, Nova Scotia and Yukon.

Phase 1 of Alberta’s Immunization Plan began on 15 December 2020, lasting three months. Frontline healthcare workers (HCWs) were the primary target group, followed by residents of long-term care facilities (LTCFs) (Phase 1A), seniors (aged 75+) and First Nations people living on reserve, Inuit and on-settlement Métis individuals aged 65+ (Phase 1B) [40]. Phase 1A included specific groups of HCWs based mainly on setting–*see S1 Table (Supporting Information)*. This prioritization strategy differed from initial NACI guidance [34]. In response, the United Nurses of Alberta published an open letter to the Minister of Health requesting all healthcare workers receive the same priority, citing, among other reasons, a disproportionate impact of the pandemic on racialized workers and newcomers who were overrepresented in frontline healthcare jobs [41]. Phase 2A divided into four elements, opened eligibility to all Albertans 65+, Indigenous individuals aged 50+ (both on- and off-reserve), and, those living and working in supportive care facilities [42]. Priority was given to those with underlying medical conditions (Phase 2B), remaining HCWs, residents and staff of congregate living facilities, and caregivers (Phase 2C), all Albertans 50+ and Indigenous individuals aged 35+ (Phase 2D) [43]. Alberta reached Phase 3 of the vaccine rollout in May 2021, opening up vaccination appointments for all Albertans 30+ on May 6, followed by full eligibility (everyone 12+) on May 10, 2021 [44].

The COVID-19 vaccine rollouts in Ontario and Nova Scotia generally reflected that in Alberta [45–47], with some notable differences. During Phase 2, Ontario prioritized younger individuals living in ‘hotspots’ (i.e., postal codes with high rates of transmission, hospitalizations and deaths) [45, 48], as well as targeted mobile vaccination of older citizens. Additionally, individuals living with underlying medical conditions were stratified by risk and prioritized accordingly during Phase 2 [45, 49]. The Ontario COVID-19 Science Table served as a prominent source of evidence for vaccine policy development in Ontario and beyond, producing regular Science Briefs that remained publicly available throughout the pandemic [50]. Of note, targeted hotspot vaccination was also recommended by Alberta Health Services (AHS) COVID-19 Scientific Advisory Group for the Phase 3 of the rollout [51] but not implemented.

Nova Scotia’s strategy focused heavily on occupation as the most significant risk factor beyond age [46]. Its Phase 2 plan featured the prioritization of healthcare workers and congregate living facilities staff, but did not include individuals living with chronic medical conditions [47]. The plan also highlighted the need to engage with First Nations and African Nova Scotian communities [47]. No hotspots were publicly announced but priority clinics were held in Indigenous communities starting in late February 2021 (Phase 1) [52] and in African Nova Scotian communities starting in April 2021 (Phase 2) [53]. The plan initially also included truckers, rotational workers and food industry workers but these groups were subsequently moved to the general age-based rollout in Phase 3 [52].

The Yukon experience was notably different. Its Phase 1 rollout began on 4 January 2021 focused on staff and residents of LTCFs as the highest priority group, followed by healthcare workers, older adults (aged 70+) and those living in shelters and correctional facilities, as well as rural and remote communities [54]. Yukon quickly moved into Phase 2 in less than one month, opening up clinics to Yukoners aged 60+ on 1 February 2021, followed by all residents aged 18+ on February 10, 2021 [55]. Phase 3 started on May 31, 2021, with vaccine eligibility opened up to everyone aged 12+ [56].

**Fig 2:**
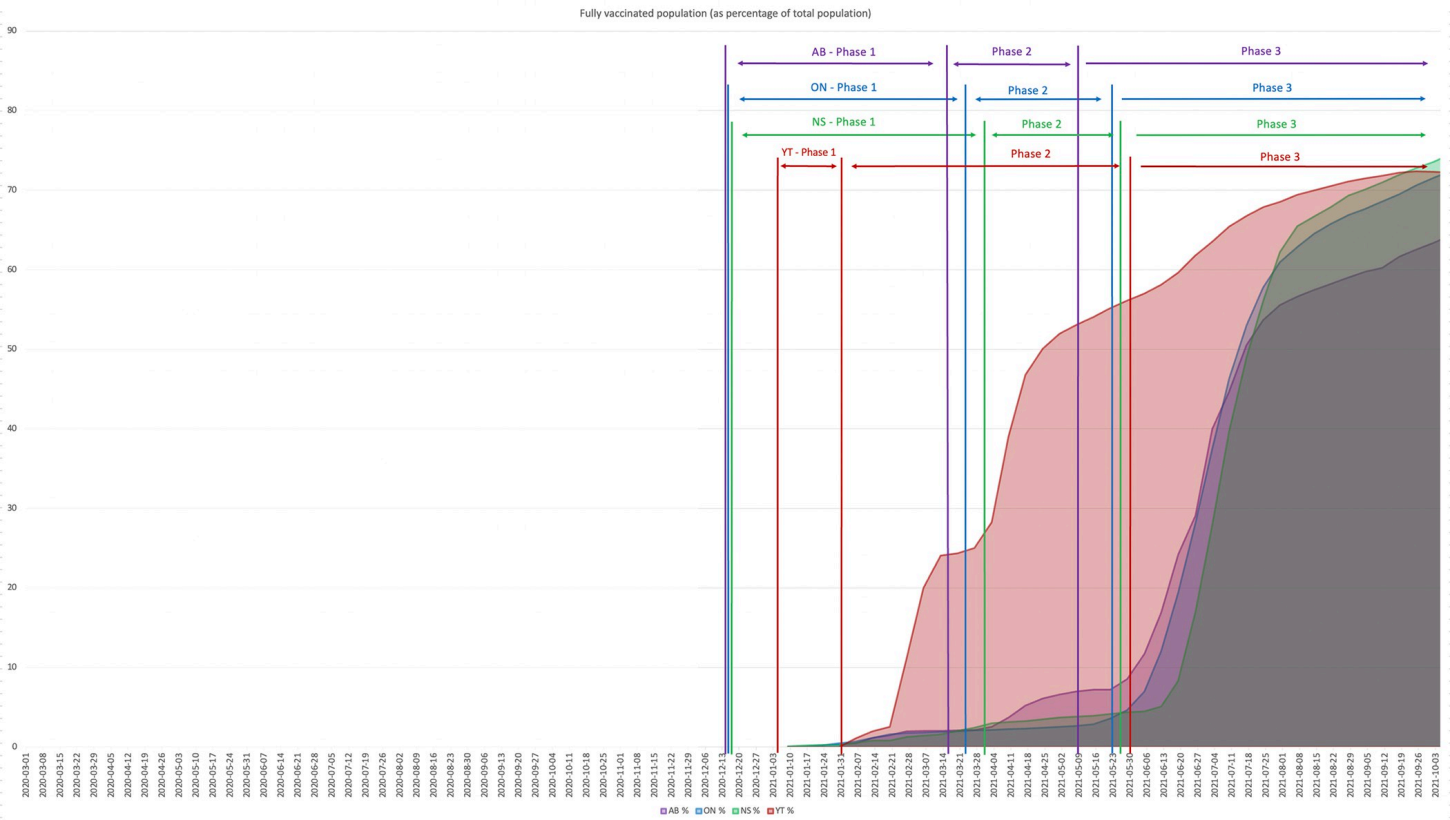
Vaccine coverage by province. COVID-19 vaccination coverage: fully vaccinated population (as a percentage of the total population) in Alberta, Ontario, Nova Scotia and Yukon (March 1, 2020 –October 5, 2021). Three phases of the vaccine rollout are indicated.

*Fig 2: Vaccine Coverage by Province* illustrates percentage of fully vaccinated in Alberta, Ontario, Nova Scotia and Yukon (from March 1, 2020, to October 5, 2021), showing the vaccine rollout phases. Alberta, Ontario and Nova Scotia followed a similar population trend for coverage, with a slow increase during Phase 1 and Phase 2, and a drastic surge within the first two months of Phase 3 (following full vaccine eligibility for aged 12+), followed by a clear slowdown. All three jurisdictions reached this point around the same time (beginning of August 2021) but with different levels of coverage; on August 7, 2021, Alberta, Ontario and Nova Scotia reported 57%, 63%, and 65% coverage respectively. Immunization efforts continued, and by October 30, 2021, 67% in Alberta, 74% in Ontario, and 77% in Nova Scotia were fully vaccinated. Yukon’s vaccination coverage showed an earlier and steadier increase throughout Phase 2 (February-May 2021) due to broader eligibility. But similar to Alberta, Ontario, and Nova Scotia, it experienced an initial increase in coverage in Phase 3, followed by a slowdown. Yukon coverage plateaued at 72% by mid-September, only increasing by 0.7% by end of October.

### COVID-19 vaccine rollouts: Beyond the numbers into the qualitative context

While the epidemiologic data, coverage rates and implementation plans paint the picture of a steady and generally successful vaccine deployment (with jurisdictional differences outlined above), key informants directly involved in vaccine rollouts identified numerous barriers that disproportionately impacted vulnerable groups across the country.

#### Phase 1: Supply and storage concerns

In the very early days of the rollout, vaccines were delivered directly to frontline healthcare workers through small immunization clinics set up next to receiving sites due to strict handling and ultra-low temperature storage requirements for the Pfizer-BioNTech vaccine [57–59]. These initial requirements contributed to the delay in vaccination in Yukon, where the rollout started some 3 weeks later with Moderna’s vaccine with more flexible cold storage requirements [54, 59]. Health Canada relaxed the requirements for the Pfizer-BioNTech vaccine on March 3, 2021, allowing more flexibility in vaccine transportation, storage, and delivery [60]. Moderna remained the main vaccine for adults in Yukon (and Nunavut and Northwest Territories) throughout the rollout, whereas in other provinces multiple vaccines were available after supply was established [37, 61].

Vaccine brand preference conversations were prominent across Canada. FL2, a primary care physician from Nova Scotia, explained:

The brand of vaccine really mattered. So, for instance, we got Moderna first, not Pfizer. And Moderna is something mostly known in Canada and North America. In a lot of people’s home countries, people didn’t know about what Moderna was. And there’s the travel restrictions started coming out [and] people started paying attention to that. They’re like, “Oh, I’m going to need to have the right brand.” Even people who wanted the vaccine wanted to get Pfizer because Pfizer was something that people [knew]. […] So, a lot of folks were like, “Moderna? […] No one back home knows what Moderna is. They all know what Pfizer is. So, Pfizer is going to be the one that’s going to let me travel.” So, a lot of folks declined Moderna because they wanted to wait for Pfizer.

In short, many Canadians, and newcomers who wished to travel internationally, considered brand and company profile, besides well-described concerns about safety and efficacy, when making vaccination decisions [61].

#### Phase 2: Priority groups and supersites

After vaccine eligibility opened to priority groups beyond healthcare workers and LTCF residents—defined by each province and typically based on age and medical or occupational risk *(see S1 Table in Supporting Information)*—‘mass’ rollouts began, primarily using large immunization ‘clinics’ (aka supersites) accessible by appointment only. FL6, a primary care physician working in Alberta, described this Phase:

I think, in the beginning, there wasn’t that awareness that some of the barriers that people were facing, like […] difficulty with transit, difficulty with booking online and literacy. And, you know, if you don’t have an Alberta Health Care number, you can’t use the online booking tool. […] I struggled in my [practice] to get people booked. It was initially rolled out by age, and then, by health comorbidity, and then, by sector. Health services workers were included first. And then, it sort of spread beyond that. So as each wave of people were eligible, it opened up further. […] But the big mass vaccine pushes were done at these big rapid access sites that they established, some of which were downtown. […] We have been telling people who live in our part of the city, “Don’t go downtown, don’t take transit, don’t go anywhere,” for a year. And then all of a sudden, it was like, now, they had to go and do this.

Large immunization clinics required conduct counter to advice given, and they had additional barriers that went largely unrecognized at the start of Phase 2. There were technological, language and human resource barriers posed by online and phone booking, as well as requirement of a provincial health card number, which made vaccines inaccessible to many newcomers and the uninsured [10]. Additionally, location, availability of appointments, and cultural safety of these sites presented barriers that were not solved by simply opening up eligibility, as S1, a global immunization policy expert, explained:

Now, if we move to some of the jurisdictions where we’ve got much more diverse ethnic populations, we know that immigrants and refugees and people who’d lived in Canada for less than five years had much lower uptake rates than people who’d been here for several generations. And part of that was reflected in the kinds of work that many of them were doing, which was […] pay for the hour, minimum wage, many of them were in essential [work]. If we look at Peel Region in Ontario, they were working as essential workers. So, it wasn’t like they could just say, “I’m out of here, okay?” Because they couldn’t. And again, originally, we didn’t go to them to make it easy for them. We didn’t give them paid time off, the ones that were working as PSWs [personal support workers]. We did not have […] the leaders in those [ethnic] communities come after them and support that for being done. […] So again, it wasn’t tailored to fit those groups.

The neglect of optimal accessibility conditions for low-paid and precariously employed essential workers who bore the brunt of the pandemic [62] can be contrasted with the significant financial supports made available to business owners [63]. The above quote also highlights the importance of culturally appropriate vaccination sites. PH11, a federal public health official, further explained the importance of cultural safety training for vaccinators from an Indigenous perspective:

This is so important, because, you know, many of our partners are telling us that they […] not just don’t trust the Western system, going to clinics or hospitals. They will go there only as a last resort because of a number of factors. […] Sometimes, it’s a culturally unwelcoming setting, people don’t understand the concerns that they have. So, they’ll just keep saying, “Why are you concerned about microchips? Why are you concerned about this as government experimentation? That’s not true.” […] One has to understand that historically, with residential schools, government has imposed this. And so … if you are from that community, you say, “Hmm, do I trust [that]?” […] We’ve been seeing that when Indigenous clients [and] patients are considering going to vaccination clinics. If there’s cultural environment inside that clinic, including Elders, ceremonies, all of that, that’s very, very helpful. It becomes a celebration…

FL2 shared his experience in Nova Scotia:

There was a very targeted effort around Indigenous populations. […] Public Health and Department of Health reached out directly to the Bands […] and said, like, “Hey, we’ve got COVID vaccines. […] You’re getting priority on it; how do you want to do it?” […] which was fantastic, right? […] That’s just exactly how you have to do it. Like, “We will support you in any way, here are the resources if you want them, but if you don’t want them, you don’t have to take them.”

Several interviewees identified focused outreach, early engagement, and partnerships with Indigenous communities as important aspects of vaccine rollout that occurred unevenly. When it did happen, successes were achieved. King et al. describe the success of a vaccine delivery model led by the Métis Nation of Alberta, which relied on local leadership exercising self-governance and culturally-safe practices [64]. The prioritization of Indigenous Peoples, when it did occur, sparked some racist discourse and conduct, [65, 66], reflecting misunderstanding of Canada’s colonial history, impact of social determinants of health and the federal government’s fiduciary responsibility for Indigenous health. Similar strategies targeted at other vulnerable communities were largely lacking in the jurisdictions examined during this Phase, with the exception of targeted outreach to African Nova Scotians [53].

#### Phase 3: Pop-up clinics and specialized settings

Once COVID-19 vaccines were made available to the general public aged 12+, appointments could be pre-booked at a variety of mass vaccination sites or accessed through pop-up clinics. While this approach was considered a success [67], FL4, a primary care physician in Ontario, cautioned uneven access continued:

In Toronto […] you’d have these pop-up clinics […]—I’m sure across the country—and lines that would go around the block. But, you know, whenever I would see them, it really impressed me how many people were [there]. I don’t know if I would stay in a three-hour line. Like, who can stay in a three-hour lineup? And people were doing it, right? They were taking time off work to do it.

As vaccine uptake increased, equity concerns were increasingly raised. FL2 elaborates:

Okay, how do we get the most people in the quickest amount of time immunized? It’s a noble thing… But I mean, […] that mindset of how we can get the most people, the easiest, is […] the impetus behind systemic discrimination. […] When we’re making a system, the easiest people to get to are people [who] generally have […] a certain level of education, who have a certain amount of income, live in certain areas, usually cities. What happens is our system slowly just gets built up in such a way that people from marginalized populations don’t get access to whatever the resource is because they’re harder to get to. And so, this system, while it sounds great, […] what ended up happening is all the English-speaking middle-income people with […] university education who lived in cities got immunized first. The people with privilege.

PH20, a public health official from western Canada, described a reluctance to attend these supersites despite high risk of transmission:

It’s hard to know the right balance, but certainly […] supersite, mass clinic approach doesn’t work for everyone. […] Even though the supersites and the booking were open to anyone, phone, online, multiple hours, […] [those] living in that first quintile of income, BIPOC groups that don’t feel as safe coming to sites like this. So, we had multiple pop-up clinics, multiple outreach clinics, in districts that had both high transmission and low vaccine rates, which tended to be those groups. So, a lot of grassroots [efforts]. We found, even though the physical access was there, there were […] still a lot of barriers to accessing that for certain populations and many of them were at high risk of transmission.

Multiple interviewees noted that grassroots community organizations and primary care providers did the work to reach vulnerable groups such as low-income racialized individuals, migrant workers, newcomers and refugees, and those who are undocumented and/or uninsured. There was limited support from provincial governments and health authorities. FL6 emphasized the difficulty of getting people to understand the barriers to access faced by these communities:

It wasn’t that they didn’t want to be vaccinated at all. It was much more that people couldn’t get to be vaccinated because perhaps they [had] three low-end jobs. […] And so, when we first—as community providers—stood up and kept saying, “Hey, we want to go deliver this, in particular, to the meat plants,” there was some […] pushback in terms of following proper process and not ruffling too many feathers. […] I think whenever these systems evolve, there’s always lots of players. […] Some of that bureaucracy was slowing things down when really it was an emergency, right? […] And now, we’re being held up as an example […] and there’s a van that goes to places and does vaccines. And even just the concept of having a drop-in concept of a clinic […] caused lots of controversy at the time but yet, lo and behold, the Premier showed up. It was all over media, everybody loves it.

FL7, a primary care physician, shared his experience providing vaccines to undocumented individuals in Ontario, many of whom had multiple intersecting vulnerabilities:

[When we] initially started the vaccines, we had long lineups in the summer, and the spring. And sometimes, the police cars would come by just because they patrol the area we’re in. And people would run from the line. They would run from the vaccine line and hide behind trees and hide behind places that couldn’t be seen. So, that was quite shocking to us […] And these are people who are all without CERB [Canada Emergency Response Benefit]. They have to go to work, […] frontline often. Mostly working in low-paying, entry-level jobs. And often working for less because their status is exploited. Plus, we had over 700 females and a couple of males in the sex trade industry on the streets and in the massage parlors. Virtually 100% of them, Asian women in our area who are being exploited. […] And people that […] both could not use [booking systems] because they wanted to know OHIP [Ontario Health Insurance Plan] number and then, wouldn’t use [it] because they were too afraid to put their names down. […] But you could walk in in some places. But who would walk in? You know, under those circumstances, and fear of exposure.

Intersecting socioeconomic, occupational, and housing factors slowed uptake in many communities with high risk of transmission. Although many barriers to access were removed (e.g., a pre-booking system change that allowed appointments without OHIP in Ontario; vaccination by primary care providers in Alberta and Nova Scotia), the time lag had critical consequences, as FL2 explains:

So, [due to the barriers] newcomers were not given access to the vaccine for the first 6–7 months. […] Once the numbers were to a certain point where they recognized they were going to have to make things accessible for newcomers, misinformation spread throughout the population. […] Now, the big pressing issue is [that] misinformation has really disseminated within the newcomer population—certain sub-cohorts of the newcomer population—and we’re having a lot of trouble now just convincing people to get the vaccine. Whereas before it was a matter of trying to get them to get access to it. Now, […] we have access to it. They’ve allowed us to have the vaccine at the clinic. But now a lot of folks are just like, “No, I’ve heard too many bad things.”

Misinformation and the lack of appropriate mechanisms to address it, especially when published in languages other than English and distributed through social networks, were presented as critical factors affecting uptake. Participants suggested the need for ongoing engagement with communities to instill vaccine confidence, along with providing a welcoming vaccination site. FL5, an infectious disease specialist from Ontario, explained:

Several strategies have been used to reduce hesitancy. […] Number one is to ensure that, wherever possible, where mass vaccinations are being done, there’s a good representation of the vaccinators coming from the communities being vaccinated. In the case of Black communities, you want a whole bunch of Black vaccinators. Number two is that you want to vaccinate in a setting where people feel culturally comfortable. […] Community centers that are connected with various diaspora groups, for example, where it’s a family affair kind of thing, it’s a special event that might be similar to other types of events that they have been to, except now a vaccine is being offered. And the atmosphere might be very different than what one would see at conventional vaccine clinic. […] There’s music, and there’s food, and there’s balloons, and there’s ice cream. And […] ensuring that there’s all the infection control measures put in place, but it is different than lining up at a convention center, or sports arena to be vaccinated.

Our public health experts identified the importance of representation among vaccinators and healthcare providers providing vaccine counselling along with diversity in decision-making and public health communication for improved trust and adherence to recommendations.

## Discussion

### Supply issues

The COVD-19 vaccine rollout in the four jurisdictions featured three phases of variable intensity and delivery models that initially relied on mass immunization sites, with targeted outreach approaches established only later in the rollout process, usually in response to community groups or frontline healthcare providers. Concerns about access during Phase 1 and Phase 2 included supply, brand recognition, and site convenience [61, 68]. The disjointed supply/delivery ‘system’ in Canada (the federal government secures supply and ships vaccines to the provinces and territories, which are then tasked with delivery) resulted in logistical challenges and delays [37]. Initial supply issues provoked tensions when the Alberta Minister of Health, for example, publicly blamed the federal government for a slow rollout [69]. Politicized blaming by some provinces of federal programs was a feature of the public response throughout the pandemic, contributing to frustration and mistrust by Canadians [70]. This mistrust was compounded by inadequate emergency preparedness and insufficient platforms for cross-jurisdictional collaboration and lesson-learning [37, 71].

### Supersite-based barriers

Delivery through large venues like sports facilities and convention centres via online or phone appointment bookings certainly facilitated rapid rollout, as demonstrated in the USA [72], but mass vaccination at supersites in the absence of parallel models fails to mitigate socioeconomic inequities and counter the vulnerabilities of equity-deserving groups [73]. Although structural racism, distrust in the healthcare system, and lack of cultural safety in medical encounters [65, 74–77] are recognized as root causes of vaccine hesitancy among Indigenous Peoples [65, 74], Black people [74, 75], people of colour [74], and newcomers [76], vaccination delivery systems were not tailored to serve these communities in an effective and timely fashion. And additional access barriers that the supersites presented (e.g., need for technology to book, time off from work, and public transportation, etc.) were not considered [10, 77].

Canadian models ensured delivery to those with easy access to booking technology, transportation, and time, and to those with health cards. For the most, these represent the least vulnerable. The experts interviewed emphasized the negative impact of the initial failure to attend to outreach and services in multiple languages, opportunities for walk-in appointments (including for those without provincial healthcare coverage), location and perceived safety of immunization sites, and cultural safety training for vaccinators as critical shortfalls. While some of these were addressed in Phase 3 of the rollouts, the delay and lack of initial engagement with vulnerable communities contributed to system distrust and hesitancy, further exacerbating existing inequalities and poorer outcomes.

### Neglecting vulnerability

Although COVID-19 epidemiology varied significantly across Canada [1], mass vaccine rollout strategies achieved high immunization rates in the general population and can be considered a success when compared to other high-income countries [67]. What gets lost in this population-based view, however, is that vaccine delivery systems largely neglected social determinants of health [13], despite their known impact on both risk of acquiring COVID-19 and access to healthcare services [2, 3, 6–10].

As evident from the vaccine uptake data, once access was granted to all aged 12+, immunization rates rose quickly before plateauing. Our analysis shows that lack of attention to vulnerability and meaningful engagement with equity-deserving communities contributed to this trend. Nova Scotia, for example, performed well during the pandemic [38], reached one of the highest vaccine coverage rates in the country (86%—as of June 19, 2022 [78]) and is overall regarded as a success story of a community coming together during a crisis [79]. Nonetheless, while Phase 2 rollout starting on April 1, 2021, made vaccines available for all people in Nova Scotia aged 70+ (regardless of health insurance and citizenship status), inconsistent and potentially discouraging messaging remained [80], as did substantial language, time, and technological barriers for bookings [81, 82]. Newcomer Health Clinic, an established community clinic that serves refugees in Halifax [83] did not receive vaccines until late June 2021 [84]. These factors together with intersecting vulnerabilities experienced by newcomers, refugees and temporary workers contributed to a critical time lag that reinforced vulnerability, fueled vaccine hesitancy and undermined trust. Vulnerability was not properly accounted for early in the vaccine rollout and barriers were only addressed vaccine uptake plateaued well into Phase 3 of the rollout.

### Conclusions and recommendations

Although Canada’s COVID-19 vaccine delivery effort has been characterized as successful [67] and may indeed have been effective and accessible for privileged members of the public and better-resourced communities, it failed to achieve equity goals. Its late (rather than parallel) and uneven engagement with vulnerable groups meant that the system contributed to vulnerability, leaving those at high risk behind by creating additional structural barriers to vaccine access. This was seen across the country, both in jurisdictions with generally well-accepted rollouts (e.g., Nova Scotia), as well as those on the other end of the spectrum (e.g., Alberta).

**Fig 3:**
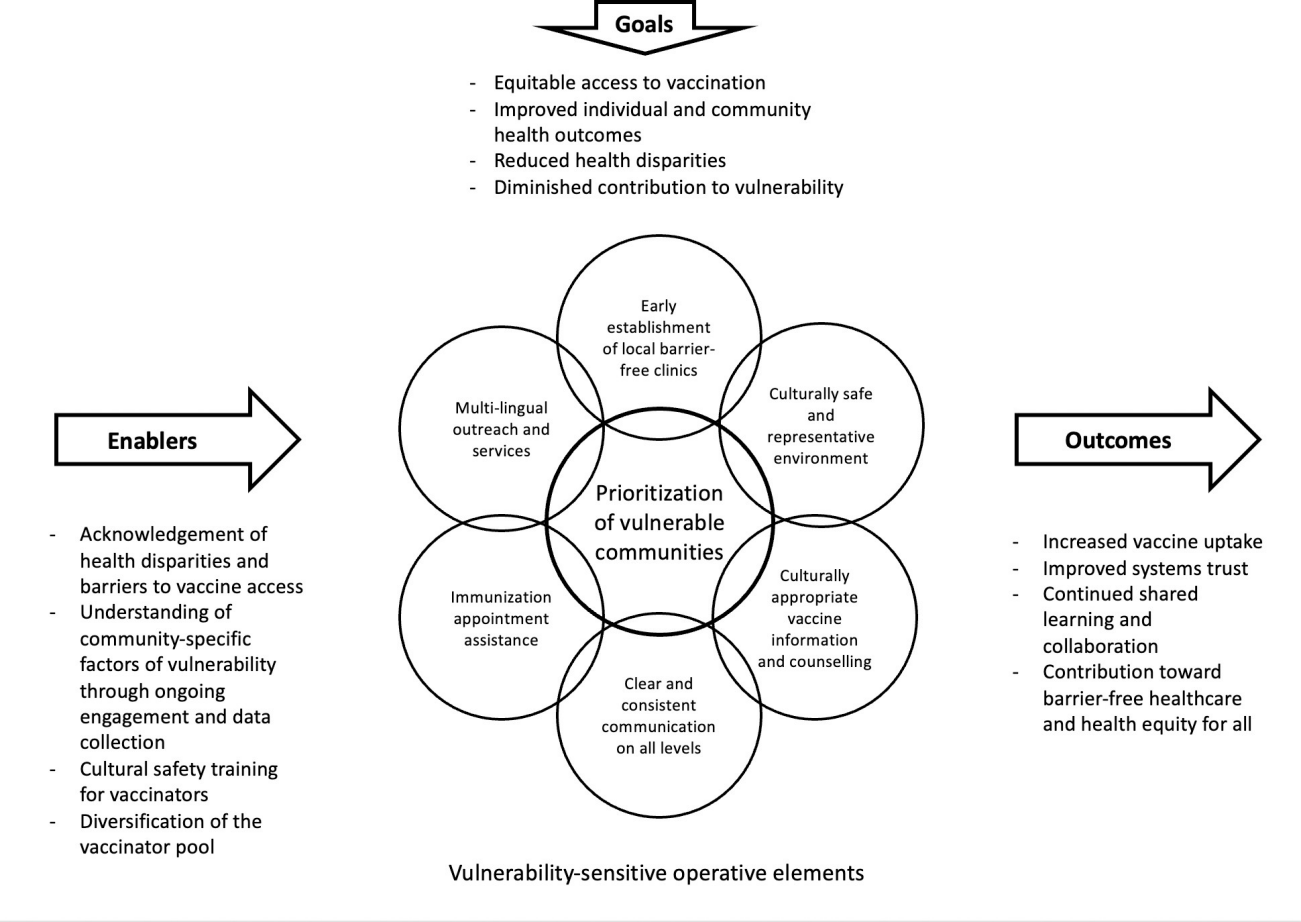
Equitable vaccine delivery model. Proposed model of an equitable vaccine delivery system. Program enablers and goals, as well as the required vulnerability-sensitive operative elements, are shown.

Based on barriers to equity identified here (i.e., supply issues, supersites, absence of vulnerability-sensitive practices), we suggest that vaccine rollout plans during public health and other emergencies must not only be ready to operationalize on short notice, but need to be much more sensitive to conditions of vulnerability within diverse communities. To achieve this improved (more equitable) operation, much more granular data (i.e., addressing the factors of vulnerability) must be collected on an ongoing basis, and processes for rapidly developing operational/deployment practices that are sensitive to those factors must be designed *(see Fig 3).*

To achieve equitable vaccine delivery, the barriers to vaccine access need to be identified and acknowledged in close consultation with the communities. Consultations have to be part of ongoing engagement and collaboration with communities, their leaders, and the grassroots organizations that already support them. Increased cultural safety training for healthcare providers and diversification of the vaccinator pool can be integral to improving infrastructure and longitudinal stabilizing of human resources of community clinics.

Importantly, a vaccine rollout model that explicitly prioritizes vulnerable communities and seeks to overcome barriers and inequities to health services should not be restricted to an emergency response plan. Routine delivery strategies must also benefit from the insights offered by recognizing the successes and failures of the COVID-19 vaccine rollout. Based on the findings of this study, we identified multiple components that are important in emergency and routine settings, such as multi-lingual outreach and services, immunization appointment assistance (e.g., transportation services, childcare), and early establishment of local barrier-free clinics (e.g., easy to access location, walk-in appointments, no provincial insurance requirements). Immunization clinics can be set up in local health centres [83, 85] and other familiar locations, such as community centres and religious venues. Healthcare providers should be trained to provide non-judgemental and culturally appropriate vaccine information and counselling to community members, communicating in a clear and consistent manner.

Notably, the goals of this vaccine delivery model go beyond improving vaccine delivery and uptake but to reduce health disparities and diminish the contribution to vulnerability. We argue that vaccine programs need to be a more integrated part of a better-supported public health system that foregrounds public health as a public good while acknowledging the complexities of intersectional realities. Reformed vaccine (and complementary public health) programs must include platforms for ongoing collaboration with the communities they serve, for evidence generation, exchange and shared learning across jurisdictions, as well as ongoing work and advocacy for barrier-free healthcare and health equity for all. Such efforts will go a long way in fostering trust in the public health and immunization systems, and in those operating within the system.

In conclusion, Canada’s mass vaccine rollout strategies did not address vulnerability and failed to reach vulnerable communities (newcomers and refugees, migrant workers, low-income racialized groups). While population level data may indicate a successful rollout, more situationally-nuanced, granular and community-specific data would suggest a very different narrative highlighting health inequities and uneven access. Vaccine delivery systems need to be improved in order to ensure more equitable access.

### Limitations

Although the data for this study were collected and analyzed using a combination of well-established methods, there were some limitations, most prominently, the lack of complete data within, between and across jurisdictions. Epidemiological and policy data were collected for four Canadian jurisdictions, while the interviewees represented nine provincial/territorial jurisdictions (with some only featuring one participant), as well as federal and global levels. Additionally, we acknowledge that our lack of personal lived experience of racialization, as well as the lack of available race-based data for COVID-19 epidemiology and vaccine status, limits our ability to fully understand to what extent the pandemic has affected racialized communities.

## Supporting information

S1 Table Prioritization of populations. Priority populations included in vaccine rollouts in Alberta, Ontario, Nova Scotia and Yukon, compared to initial NACI recommendations (published in November 2020).
(DOCX)
