# Supplementary material for: An equitable vaccine delivery system: Lessons from the COVID-19 vaccine rollout in Canada
Source: PLoS One. 2022 Dec 30;17(12):e0279929. doi: 10.1371/journal.pone.0279929 (PMC9803301; doi:10.1371/journal.pone.0279929)
Supplement: S1 Table — Priority populations included in vaccine rollouts in Alberta, Ontario, Nova Scotia and Yukon, compared to initial NACI recommendations (published in November 2020). (DOCX) [file pone.0279929.s001.docx]

**Supporting Information**

*Table 1. Prioritization of Populations.* Priority populations included in vaccine rollouts in Alberta, Ontario, Nova Scotia and Yukon, compared to initial NACI recommendations (published in November 2020).

| **NACI-Recommended Priority Group** | **Vaccine Access Granted** | | | |
| --- | --- | --- | --- | --- |
|  | **Alberta** | **Ontario** | **Nova Scotia** | **Yukon** |
| Those at high risk of severe illness and death:   - Advanced age - High-risk health conditions (as defined by jurisdictional governments) | 1B (Feb 24): 75+  2A (Mar 15): 65+  2D (April 30): 50+  2B (Mar 30): 62+  2B (April 5): 58+  2B (April 23): 16+ | 1 (Mar 15): 80+  2 (Mar 22): 75+  2 (Mar 27)^[[1]](#endnote-1)^: 70+  2 (April 2)^[[2]](#endnote-2)^: 60+  2 (Apr 30^[[3]](#endnote-3)^): 55+  2 (May 3): 50+  2 (April 27): Highest risk^[[4]](#endnote-4)^  2 (May 3): High risk  2 (May 10): At risk | 1 (Feb 22): 80+  1 (Mar 26): 75+  2 (April 1): 70+  2 (April 19): 60+  2 (April 26): 55+  2 (May 2): 50+  N/A | 1 (Jan 4): 80+  2 (Jan 20)^[[5]](#endnote-5)^: 70+  2 (Feb 1)^[[6]](#endnote-6)^: 60+  N/A |
| Those most likely to transmit COVID-19 to those at high risk and workers essential for COVID-19 response:   - LTCF workers and residents - Frontline HCWs - Congregate living facilities staff and residents - Household contacts of those at high risk | 1A (Dec 15)  1A (Dec 15)^[[7]](#endnote-7)^  2C (April 12)^[[8]](#endnote-8)^  2C (April 30)  2C (April 30) | 1 (Dec 15)  1 (Dec 15)^[[9]](#endnote-9)^  2 (April 27)  2 (April 27) | 1 (Dec 16)  1 (Dec 16)^[[10]](#endnote-10)^  1 (Mar 2)^[[11]](#endnote-11)^  1 (Jan 5)  N/A | 1 (Jan 4)  1 (Jan 4)  2 (Jan 20)  N/A |
| Those contributing to the essential services (as defined by jurisdictional governments) | 2C (April 30): First responders | 2 (April 29): Licensed childcare workers  2 (May 3): Workers who cannot work from home – Group 1^[[12]](#endnote-12)^  2 (May 10): Group 2^[[13]](#endnote-13)^ | 2 (Mar 2): First responders^[[14]](#endnote-14)^ | N/A |
| Those whose living or working conditions put them at elevated risk and where infection could have disproportionate consequences:   - Indigenous communities - Others (as defined by jurisdictional governments) | 1B (Feb 24): 65+ on-reserve only  2A (Mar 15): 50+ on and off-reserve  2D (April 30): 35+ on and off-reserve  N/A | 1 (Feb 1): 18+ in Northern remote and high-risk communities (on-reserve and urban)  2 (April 27): 45+ in hotspots^[[15]](#endnote-15)^  2 (May 3): 18+ in hotspots | 1 (Feb 24): 55+ on and off-reserve  2 (April 23): 16+ on and off-reserve  2 (April 8): African Nova Scotians 55+ | 2 (Jan 20): 18+ in rural and remote communities  N/A |

1. Prioritization by region: March 27: Toronto; March 29: priority regions, including Ottawa, Peel, Hamilton, Kingston, Niagara, York etc.; April 2: Peterborough, Eastern Ontario, Haliburton, Sudbury, Thunder Bay etc. [↑](#endnote-ref-1)
2. Also following regional prioritization, starting with Toronto and Peel. [↑](#endnote-ref-2)
3. Pfizer and Moderna vaccines. AstraZeneca vaccination was performed according to a separate schedule at pharmacies and primary care units. [↑](#endnote-ref-3)
4. As determined by Ontario Ministry of Health. [↑](#endnote-ref-4)
5. Priority areas: Whitehorse, Ibex Valley, Marsh Lake and Mount Lorne areas [↑](#endnote-ref-5)
6. Whitehouse and surrounding areas. [↑](#endnote-ref-6)
7. Specific groups of HCWs: respiratory therapists, intensive care physicians and staff, emergency room physicians and staff, and long-term care and designated supportive living facility staff, home care staff. [↑](#endnote-ref-7)
8. All HCWs providing direct in-person care to patients in all settings. [↑](#endnote-ref-8)
9. Frontline workers in specific settings (including HCWs and support staff): intensive care, emergency departments, COVID-19 units, rapid response teams, COVID-19 testing centres, immunization clinics, laboratory services; first responders; home care workers; community HCWs working in specialized settings, such as Indigenous health centres or needle exchange programs. [↑](#endnote-ref-9)
10. COVID-19 units, emergency departments, critical care units, birth units, regional care units. [↑](#endnote-ref-10)
11. Community healthcare providers. [↑](#endnote-ref-11)
12. School staff, first responders, agricultural and farm workers, food manufacturing workers etc. [↑](#endnote-ref-12)
13. Workers in retail, social services, transportation, energy, waste management, mining, oil and gas industry etc. [↑](#endnote-ref-13)
14. In addition, rotational workers, truck drivers, food industry workers were initially included in Phase 2 but were moved to Phase 3 following the change in the interval between first and second doses of the vaccine. [↑](#endnote-ref-14)
15. 114 high-risk neighbourhoods identified by a postal code. [↑](#endnote-ref-15)
